# In Vitro Evaluation of Some Types of Ferrimagnetic Glass Ceramics

**DOI:** 10.1155/2014/415854

**Published:** 2014-05-08

**Authors:** S. A. M. Abdel-Hameed, M. A. Marzouk, R. L. Elwan

**Affiliations:** Glass Research Department, National Research Center, Dokki, Cairo 12311, Egypt

## Abstract

The present study aimed at studying the acceleration of the bioactive layer on the surface of ferrimagnetic glass ceramic with a basic composition 40Fe_2_O_3_–15P_2_O_5_–20SiO_2_–5TiO_2_ through the addition of 20% of different types of metal oxides like MgO or CaO or MnO or CuO or ZnO or CeO_2_. SEM, EDAX, and ICP were applied to present the results of the study. SEM and EDAX measurements indicated the presence of apatite layer formed on the surface of the prepared glass ceramics after immersion in SBF within 7 to 30 days. The investigation of the results clarified that the addition of CaO or ZnO accelerated the formation of apatite on the surfaces of the samples in the simulated body fluid faster than other metal oxides. Inductive coupled plasma (ICP) analysis shows the evolution of ion extraction by the simulated body fluid solution (SBF) with time in relation to the elemental composition.

## 1. Introduction

Bioactive materials [1, 2] are known to bond to living bone in the body through formation of an apatite layer on their surface [3]. Bioactive glasses and glass ceramics find a variety of biological applications such as in cell separation, drug delivery, and magnetic intracellular hyperthermia treatment of cancer [4–6]. The success of bioglass [7], ceravital [8], A-W glass ceramic [9], and dense hydroxyapatite ceramic [10] materials in clinical applications [11] has propelled the search for bioactive glasses and glass ceramics with enhanced properties. Recently, development of bioglass ceramics containing a magnetic phase has received attention for application as thermoseed in hyperthermia treatment of cancer [9–11]. The interest in these materials started when SiO_2_-CaO-P_2_O_5_ glasses containing iron oxide were found to yield ferrimagnetic glass ceramics upon heat treatment at elevated temperatures [10]. The magnetic properties of this bioceramic arise from magnetite (Fe_3_O_4_) crystallized from hematite present in the glass during heat treatment. When this bioceramic is placed in the region of the tumor and is subjected to an alternating magnetic field, heat is generated by magnetic hysteresis loss [11]. The tumor is effectively heated and selectively destroyed when local temperatures of 42–45°C are attained by this process [12–14]. Magnetic glass ceramics with zinc ferrite in a CaO-SiO_2_ glass matrix [12], Fe_3_O_4_ in Na_2_O-CaO-SiO_2_-P_2_O_5_ glass matrix [13–16], and Fe_3_O_4_ in a CaO-SiO_2_ glass matrix [17] have also been developed for this purpose. Thus, the ferromagnetic bioactive glass ceramics can be used not only for the hyperthermia treatment of cancer but also as a substitute for a cancerous/damaged bone. A number of ferromagnetic glass ceramic systems have been developed for this purpose like FeO-Fe_2_O_3_-CaO-SiO_2_ [18], ZnO-Fe_2_O_3_-CaO-SiO_2_ [19], SiO_2_-Na_2_O-CaO-P_2_O_5_-FeO-Fe_2_O_3_ [20], Li_2_O-MnO_2_-CaO-P_2_O_5_-SiO_2_ [21], Fe_2_O_3_-CaO-ZnO-SiO_2_-B_2_O_3_ [22], magnetite/hydroxyl apatite composite [23], and CaO-SiO_2_-P_2_O_5_-Na_2_O-Fe_2_O_3_ [9, 10]. Bioactivity tests have shown that apatite forming ability increases in CaO-SiO_2_-P_2_O_5_-Na_2_O glasses with an increase in Fe_2_O_3_ content with proper choice of constituent compositions [24].

In previous work [25], we applied heat treatment to study the crystallization behavior and magnetic properties of ferromagnetic glass ceramics in the system Fe_2_O_3_·CaO·ZnO·SiO_2_. Another systematic study was applied to investigate the phase formation, microstructure, and both the amount and grain size of the crystallized magnetite after gradual addition of different types of nucleating agents to ferromagnetic glass ceramics [26].

The aim of this work was to evaluate the bioactivity of ferrimagnetic glass ceramics in the system Fe_2_O_3_-TiO_2_-P_2_O_5_-SiO_2_ with the addition of different types of metal oxides (MgO-CaO-MnO-ZnO-CeO_2_-CuO). The bioactivity was evaluated in vitro by examining the formation of bone-like apatite layer on their surfaces when treated in simulated body fluid (SBF).

## 2. Experimental Procedures

### 2.1. Glass Composition and Preparation

The glass ceramics were prepared from dry mixtures of reagent grade SiO_2_, Fe_2_O_3_, TiO_2_, and P_2_O_5_ with the batch composition 40Fe_2_O_3_-15P_2_O_5_-20SiO_2_-5TiO_2_ in addition to 20% changeable metal oxides (CaO as CaCO_3_, MnO, MgO, CeO_2_, CuO, and ZnO). Our target was to obtain a glass ceramic, not a ceramic material, so a melting step was necessary to achieve the nucleation of magnetite in a liquid-derived amorphous phase. The batches were placed in a platinum crucible and melted in an electric furnace at 1450°C for 2 h. The melts were poured onto a stainless steel plate at room temperature and pressed into a plate 1-2 mm thick by another cold steel plate.

### 2.2. Soaking in the Simulated Body Fluid (SBF)

Each sample was soaked in a cellular simulated body fluid (SBF, 50 mL), with ion concentrations and pH nearly equal to those of human blood plasma [17] as shown in Table 1. The SBF was prepared according to Kokubo [1] by dissolving reagent grade NaCl, NaHCO_3_, KCl, K_2_HPO_4_-3H_2_O, MgCl_2_-6H_2_O, CaCl_2_, and Na_2_SO_4_ in an ion exchanged water contained in a polystyrene bottle. These reagents were added in the order listed. The solution was buffered at pH value *≈*7.25 with 50 mM of Tris-(hydroxymethyl)-amino methane ((CH_2_OH)_3_CNH_2_, hereafter as TRIS) and 45 mM hydrogen chloride and its temperature was kept at ±37°C. The soaking was carried out at 37°C, under continuous stirring and for various times (0, 15, and 30 days).

### 2.3. Measurement of Element Concentrations

After the specimens were removed from the soaking solutions, changes in the concentrations of calcium and phosphorous of these solutions were measured by inductively coupled plasma (ICP) spectrometer, equipped with ultrasonic useful for trace elements.

### 2.4. Surface Structure Analysis and Characterization

Scanning electron microscopy (SEM) and EDAX techniques were performed on the glasses before and after soaking in SBF solution. The samples were removed from this solution, washed with pure acetone, and dried at room temperature.

The morphological analyses were carried out by means of SEM technique: the microscope was equipped with an energy dispersive X-ray analyser for compositional study. The samples were mounted on a stainless steel stub using double stick tape and then coated with a thin layer of Au.

## 3. Results and Discussion

### 3.1. Morphological and Spectra Properties

**Figure 1:**
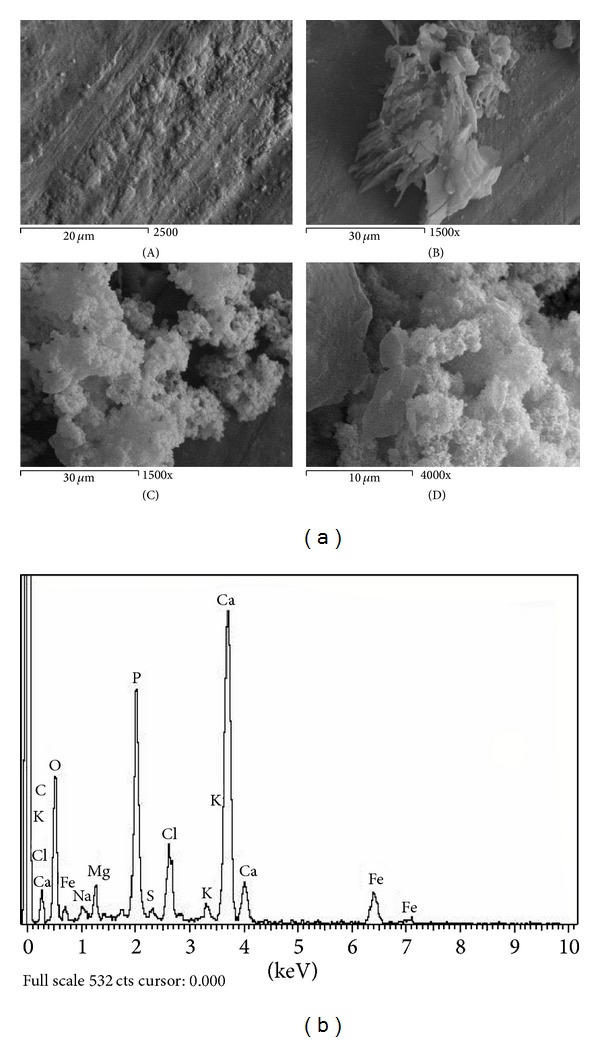
(a) SEM micrograph of Mg-doped glass ceramics (A) before, (B) after 1 week, (C) after 2 weeks, and (D) after 4-week immersion in SBF. (b) EDAX of Mg-doped glass ceramics after 4-week immersion in SBF.

**Figure 2:**
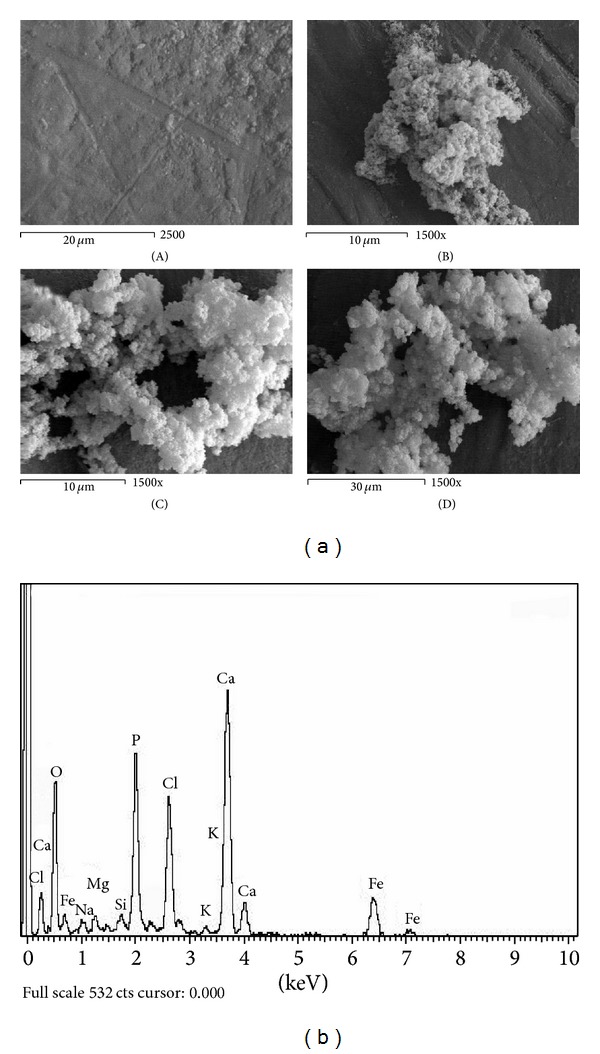
(a) SEM micrograph of Ca-doped glass ceramics (A) before, (B) after 1 week, (C) after 2 weeks, and (D) after 4 weeks of immersion in SBF. (b) EDAX of Ca-doped glass ceramics after 4-week immersion in SBF.

**Figure 3:**
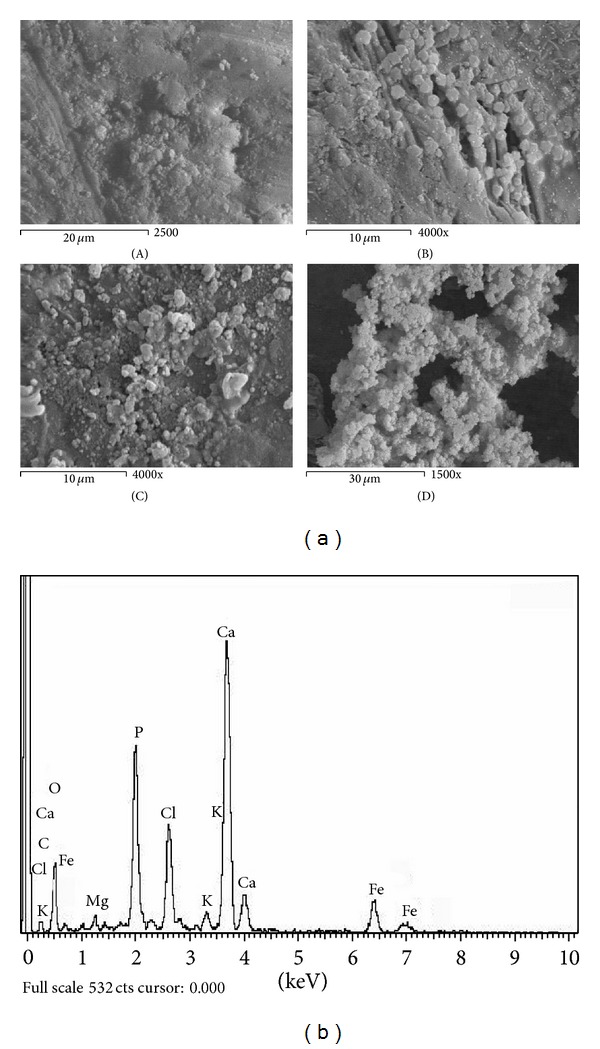
(a) SEM micrograph of Mn-doped glass ceramics (A) before, (B) after 1 week, (C) after 2 weeks, and (D) after 4 weeks of immersion in SBF. (b) EDAX of Mn-doped glass ceramics after 4-week immersion in SBF.

**Figure 4:**
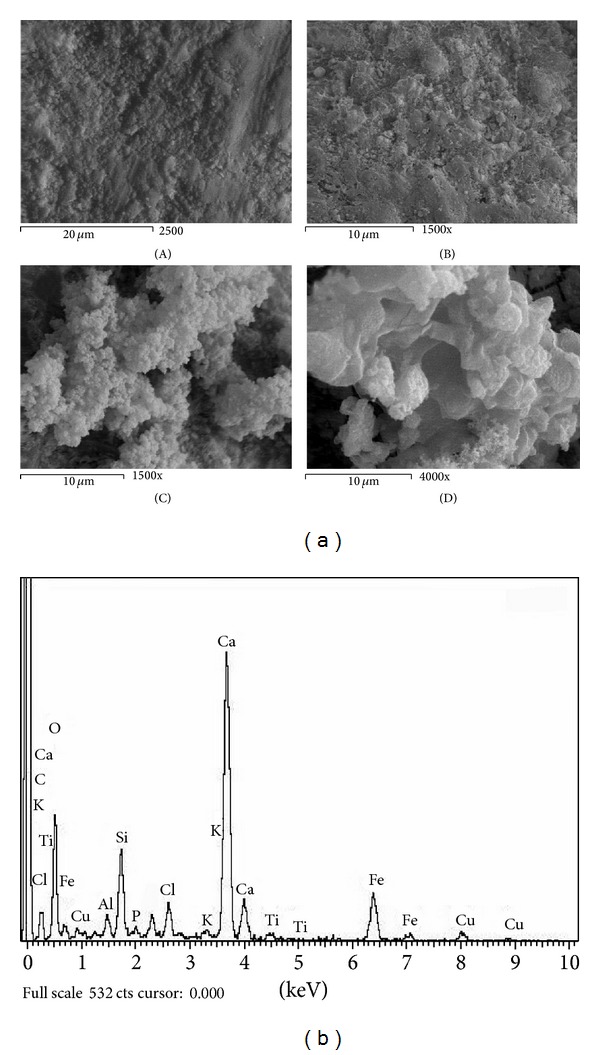
(a) SEM micrograph of Cu-doped glass ceramics (A) before, (B) after 1 week, (C) after 2 weeks, and (D) after 4 weeks of immersion in SBF. (b) EDAX of Cu-doped glass ceramics after 4-week immersion in SBF.

**Figure 5:**
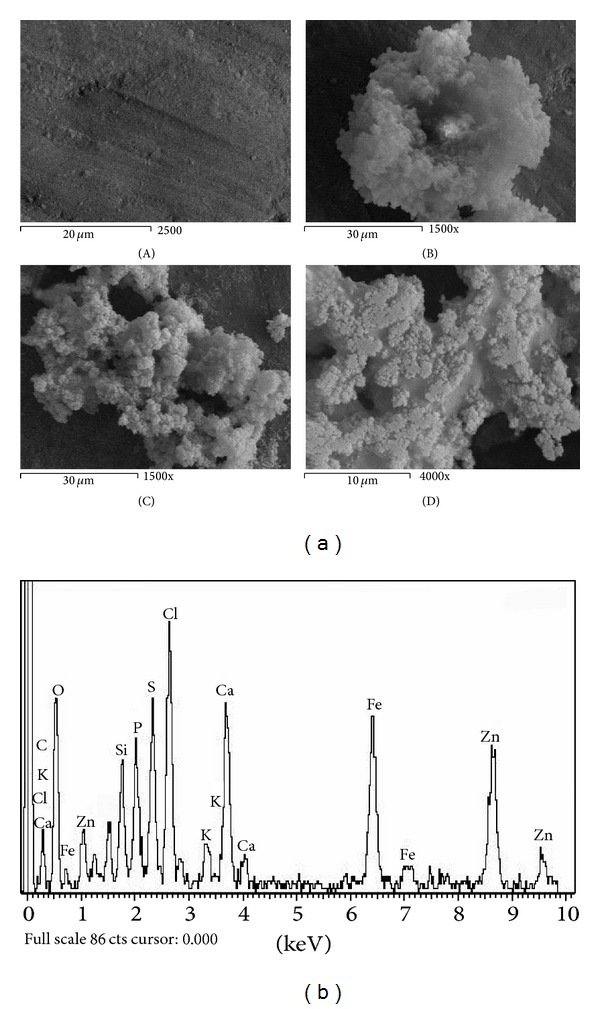
(a) SEM micrograph of Zn-doped glass ceramics (A) before, (B) after 1 week, (C) after 2 weeks, and (D) after 4 weeks of immersion in SBF. (b) EDAX of Zn-doped glass ceramics after 4-week immersion in SBF.

**Figure 6:**
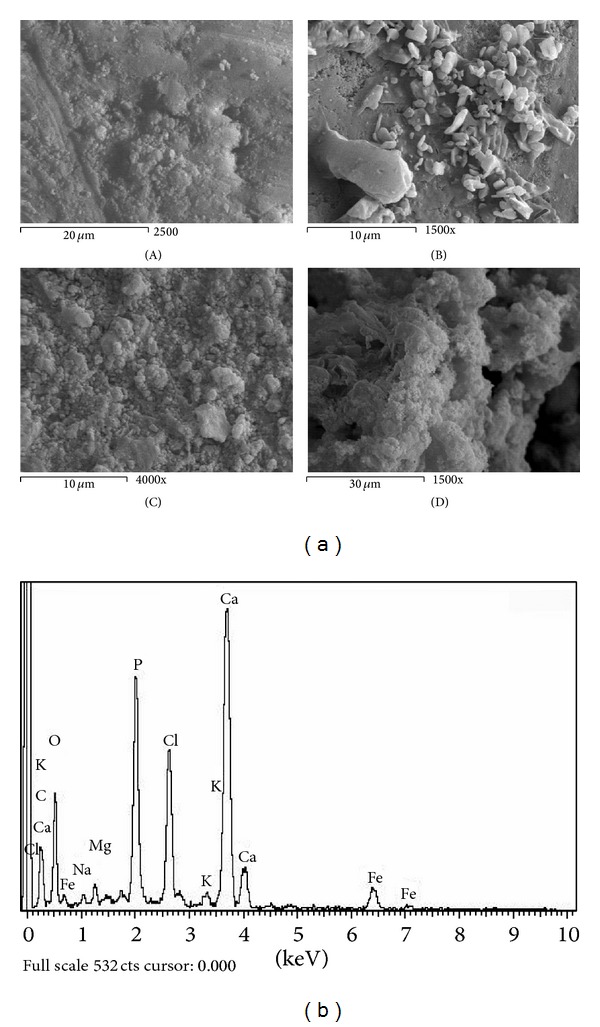
(a) SEM micrograph of Ce-doped glass ceramics (A) before, (B) after 1 week, (C) after 2 weeks, and (D) after 4 weeks of immersion in SBF. (b) EDAX of Ce-doped glass ceramics after 4-week immersion in SBF.

Figures 1(a)–6(a) show the SEM micrographs of the present series of glass ceramic of the system Fe_2_O_3_-TiO_2_-P_2_O_5_-SiO_2_ after immersion in SBF for 0, 1, 2, and 4 weeks. Increase in bioactivity with increasing zinc oxide content was observed. The results have been used to understand the evolution of the apatite surface layer as a function of glass composition and immersion time in SBF as follows.

1. It can be seen from Figures 1(a)–6(a) that combination of metal oxides (Mg, Ca, Cu, Mn, Zn, and Ce) with the basic composition produces no apatite formation on the surface before the immersion in the simulated body fluid (0 week). On the other hand, within 1–4 weeks of immersion, the apatite layer was clearly observed.
2. It was observed that the acceleration of forming the apatite layer within the early period depends on the type of the metal ion. Figures 1(a), 2(a), 5(a), and 6(a) show that Ca, Mg, Zn, and Ce accelerate the formation of apatite layer within one week while both Mn- and Cu-doped samples (Figures 3(a) and 4(a)) accelerate the formation of apatite layer within 2–4 weeks.
3. Ca- and Zn-doped glass ceramic exhibit the best results among the glasses tested in vitro in the SBF solution.
4. The EDAX analysis confirmed the formation of apatite (Figures 1(b)–6(b)).

These results might be interpreted as follows: apatite formation on the surfaces of the heat-treated glasses in the simulated body fluid is governed by the chemical reaction of the surface of the matrix with the fluid [17]. In the present series of glass ceramics, the major constituent is Fe_2_O_3_ and the addition of SiO_2_ and P_2_O_5_ in the matrix allows the formation of bioactive ferrimagnetic bioglass ceramics [17–24]. The previous assumption is confirmed by Singh and Srinivasan [27]; in their study, they assumed that the addition of metal ions to ferrimagnetic bioglass ceramics (weight ratio) accelerated the formation of apatite on the surfaces of the glasses in the simulated body fluid by increasing the pH of the surrounding fluid due to the dissolution of M^n+^ ions.

In the present case, apatite formation on the surface of the glass ceramics was accelerated by the addition of both CaO and ZnO. Ebisawa et al. [17] postulated the formation mechanism of an apatite-like layer on materials and assumed that this might be due to the release of Ca^2+^ ions, which results in the rapid increase in the pH of the solution. In such materials, an exchange of ions Ca^2+^ of material with H_3_O^+^ of the SBF is produced. The Ca^2+^ dissolved from the glass ceramics increases the Ca^2+^ concentration in the SBF, and the consumption of H_3_O^+^ results in the increase of pH value [27]. Similarly, the addition of ZnO to the present basic composition is also assumed to accelerate the formation of apatite on the glass ceramics. Zinc ions migrate into the fluid as Zn(OH)_2_ contributes more OH^−^ ions required for the crystallization of the amorphous Ca-P from the solution to form the apatite layer [27].

### 3.2. Corrosion of**  **Biosamples in SBF (Solution Analysis)

ICP analysis shows the evolution of ion extraction by the simulated body fluid solution (SBF) with time in relation to the initial elemental composition in the prepared samples. It was observed that a marked release of Ca and P ions takes place, which governs, by the bioactivity behavior of the glass, the effect of ions releasing on pH value of the solution and the composition of the bioactive glass.

The corrosion behavior data of the studied samples are exhibited in Table 2, which shows the concentrations of calcium and phosphorous species presented in the SBF solution. From Table 2, one can deduce that by soaking the samples separately in SBF, the concentrations of both Ca and P increase as they dissolve rapidly from the bulk glass samples; then, they were consumed for HCA layer formation on the glasses surfaces; by continuous immersion in the solution, all the samples dissolved and lead to higher concentrations of both Ca and P in the solution; also Table 2 shows the effect of adding different metal ions on the HCA layer formation through the difference in Ca and P concentrations in the SBF solution.

Table 2 exhibits the concentration values of Ca and P in SBF solution after soaking the samples which contain one of the following metal oxides: CaO, MnO, MgO, CeO_2_, CuO, and ZnO for 7, 14, and 30 days; from that table, we can find that by adding CaO or ZnO to sample the bioactivity of samples is increased and it accelerates HCA layer formation through the fast consuming of Ca and P ions.

The quantitative analysis of the Ca and P ions during and after SBF immersion tests is important to understand the kinetics of surface reaction in bioactive materials. The decrease or increase in Ca and P concentrations with glass soaking in SBF solution is consistent with the formation and growth or inhibition of Ca-P_2_O_5_ layer on the glass surface.

A quantitative analysis of the ions in the solution after in vitro tests is very useful to complement the understanding of surface kinetic reactions in bioactive materials [28]. The data recorded in Table 2 revealed that the concentrations of calcium and phosphorous elements in SBF solutions of glass samples decreased or increased with the ability of the glass samples to form HCA layer on their surfaces and that consequently affects their bioactive behavior. In the early stage of immersion (2 days), the concentrations of Ca, P, and Si ions were increased in the SBF solution. These results suggested that a relative interaction between SBF solution and the glass samples occurred. This may be due to the release of alkali and alkaline earth ions, loss of soluble SiO_2_ from the surface of the glass specimens to the SBF solution, condensation, and repolymerization of SiO_2_-rich layer [29]. Calcium and phosphorous concentrations then fell in the SBF solution with increasing the silicon ion concentration after 7-day duration, and this may be due to the formation of amorphous CaO-P_2_O_5_ layer on the surface of the glass sample. With increasing the silicon, the subsequent rapid decrease in phosphorous and calcium ion concentrations in SBF solution after 14-day immersion indicated that crystallization and growth of the CaO-P_2_O_5_ rich layer occurred [30]. Studies of carbonate containing hydroxy-carbonate apatite (HCA) layer formed on the surface of bioactive glasses have shown that the reactions occur on the material side in five stages. These stages are faster for the highest level of bioactivity [29].

## 4. Conclusion

Homogeneous glass ceramics of the chemical composition 40Fe_2_O_3_-15P_2_O_5_-20SiO_2_-5TiO_2_ doped with 20 mol% of varieties of metal ions like MgO, CaO, MnO, CuO, ZnO, and CeO_2_ were obtained. In vitro analysis was applied to show the bioactivity of the samples after immersion in the simulated body fluid. SEM and EDAX studies show that the prepared glass ceramics behave as a bioactive material and the formed apatite-like layer on the surface accelerated depending on the type of the metal oxide. Inductive coupled plasma (ICP) analysis shows the evolution of ion extraction by the simulated body fluid solution (SBF) with time in relation to the elemental composition. The combination of metal oxides (Mg, Ca, Cu, Mn, Zn, and Ce) with the basic composition produces no apatite formation on the surface before the immersion in the simulated body fluid (0 week), while within 1–4-week immersion the apatite layer was clearly observed. The formation of the apatite layer depends on both time of immersion and type of the metal ion. Ca-, Mg-, Zn-, and Ce-doped samples accelerate the formation of apatite layer within one week, while Mn- and Cu-doped samples accelerate the formation of apatite layer within 2–4 weeks. Ca- and Zn-doped glass ceramics exhibit the best results among the glasses tested in vitro in the SBF.

## Figures and Tables

**Table 1:** Ion concentrations of simulated body fluid and human blood plasma [17].

	Concentration (mM)							
	Na^+^	K^+^	Mg^2+^	Ca^2+^	Cl	HCO_4_^2^	HPO_4_^2^	SO_4_^2^
Simulated body fluid	142.0	5.0	1.5	2.5	147.0	4.2	1.0	0.5
Human blood plasma	142.0	5.0	1.5	2.5	103.0	27.0	1.0	0.5

**Table 2:** ICP results.

Sample	Time/weeks	Si	Ca	P	Fe
Mg	1	0.129	44.74	2.08	—
	2	—	0.310	0.032	0.21
	4	1.080	103.99	0.014	0.01

Ca	1	0.137	48.430	2.05	—
	2	—	45.97	0.023	0.03
	4	0.010	60.64	0.07	0.08

Mn	1	0.131	49.66	2.88	—
	2	—	57.54	3.455	0.11
	4	0.770	—	0.66	0.02

Cu	1	0.157	56.52	6.08	—
	2	—	35.93	0.015	0.09
	4	0.610	89.31	0.002	0.05

Zn	1	0.124	54.16	2.17	—
	2	—	33.65	1.68	0.01
	4	3.310	51.621	0.010	0.02

Ce	1	0.322	45.02	1.39	—
	2	—	66.40	4.76	2.29
	4	0.120	78.03	0.09	0.04
